# Robust Estimation and Optimized Transmission of 3D Feature Points for Computer Vision on Mobile Communication Network

**DOI:** 10.3390/s22218563

**Published:** 2022-11-07

**Authors:** Jin-Kyum Kim, Byung-Seo Park, Woosuk Kim, Jung-Tak Park, Sol Lee, Young-Ho Seo

**Affiliations:** Department of Electronic Materials Engeering, Kwangwoon University, Seoul 01897, Korea

**Keywords:** 3D keypoint, SIFT, stereo matching, scene change

## Abstract

Due to the amount of transmitted data and the security of personal or private information in wireless communication, there are cases where the information for a multimedia service should be directly transferred from the user’s device to the cloud server without the captured original images. This paper proposes a new method to generate 3D (dimensional) keypoints based on a user’s mobile device with a commercial RGB camera in a distributed computing environment such as a cloud server. The images are captured with a moving camera and 2D keypoints are extracted from them. After executing feature extraction between continuous frames, disparities are calculated between frames using the relationships between matched keypoints. The physical distance of the baseline is estimated by using the motion information of the camera, and the actual distance is calculated by using the calculated disparity and the estimated baseline. Finally, 3D keypoints are generated by adding the extracted 2D keypoints to the calculated distance. A keypoint-based scene change method is proposed as well. Due to the existing similarity between continuous frames captured from a camera, not all 3D keypoints are transferred and stored, only the new ones. Compared with the ground truth of the TUM dataset, the average error of the estimated 3D keypoints was measured as 5.98 mm, which shows that the proposed method has relatively good performance considering that it uses a commercial RGB camera on a mobile device. Furthermore, the transferred 3D keypoints were decreased to about 73.6%.

## 1. Introduction

Generating 3D keypoints is an essential technique in computer graphics and vision. Feature extraction and 3D keypoint generation can be used in many applications, such as object pose estimation, reconstruction, object or space matching, and segmentation. In addition, 3D keypoints can be used for interactive services in AR (augmented reality), VR (virtual reality), and XR (extended reality) based on these applications.

Research to find the 3D features of objects has been conducted for a long time. Initially, studies seeking to extract the features of 3D objects dealt with 3D data such as point clouds, meshes, and depth images. This led to many kinds of research, including 3D Harris [1], HKS [2], Salient Points [3], Mesh Saliency [4], Scale-Dependent Corners [5], CGF [6], and SHOT [7]. Approaches such as these extract a local descriptor for a geometric feature of the local reference frame. Because they consider only local geometric information, there are differences following 3D feature extraction. Recently, deep learning-based methods and deep functional dictionaries [8] have been developed for detecting keypoints. These methods have weaknesses in circumstances involving rotation, and S2CNN [9] and PRIN [10] have subsequently been proposed to overcome this issue.

Estimation of 3D keypoints using 2D RGB images has been studied as well. Occlusion-Net, a model for classifying 2D keypoints according to depth, is one such proposal [11], and 2D3D-MatchNet, a study to match the descriptor of 2D images with 3D keypoints of a point cloud captured by a 3D sensor, has been presented as well [12,13]. However, the deep learning-based methods have weaknesses for various input images because of their dependence on correct labels in training. It is important to consistently reflect the local features of the image, rather than always finding the same location for the keypoint. Consistency in finding features should always be maintained, even if the image is slightly changed.

Recently, various services for AR, VR, and XR services have been actively developed. Recognizing 3D space and object features is essential for these applications [14]. However, users do not have a device and method to capture 3D information directly. Therefore, a way to estimate 3D keypoints on 2D images using a camera installed in a user’s smartphone is required. In the case of practical service, an additional consideration for several issues with 3D keypoint detection is needed, including security [15], computing power [16], and the amount of transmitted data [17]. Transferring images captured by unadmitted users to detect 3D keypoints in a cloud server is a legal problem [14,18]. In avoiding this problem, however, operating all processes on the user’s device encounters limitations in terms of computing power. Generating and storing all 3D keypoints of all frames is not reasonable on the transferring side. Here, we avoid security problems by moving the 2D keypoint, not the original images. Furthermore, the computing power is distributed by performing only the amount of computation that is needed to solve the security problem on the mobile device and the rest on the server. The overlapped keypoints are not stored, and only new ones are used to update the keypoint database. Operational continuity is assured by scene change detection.

Scene change detection is widely used in applications involving video sequence segmentation for video coding, scene searching in videos, and video filtering for copyright protection [19,20,21]. The methods used to detect scene changes can be classified into three categories. The first are methods that use rules followed by media producers in scene production; while these may enhance the results of scene change detection, they encounter problems that limiting them to movies and drama [22]. The second group consists of stochastic methods, which train probability variables using the Hidden Markov model and the Markov Chain Monte Carlo Model after clustering color, edge density, and direction of a scene. This approach detects scene changes by estimating the posterior probability with the maximum value. Because this depends on the data used in training, a large amount of training data are required [23]. The final approach involves method for classifying scenes by modeling data in a graph in order to calculate, cluster, and arrange the similarity between frames and then detect scene changes using a graph segmentation algorithm [24]. There are various methods that can calculate frame similarity. This paper uses descriptor matching for keypoints on each frame, and tries to obtain frame similarity.

Approaches for end-to-end learning of 3D keypoints have been investigated previously [14]. Prior studies can be largely divided into methods of extracting 3D keypoints from a 2D images and methods of extracting 3D keypoints from a 3D model (i.e., a mesh or point cloud). Recently, various end-to-end models using deep learning have been proposed to generate 3D keypoints [12,25,26,27,28,29,30,31,32]. Zhou et al. proposed a novel unsupervised domain adaptation technique for the task of 3D keypoint prediction from a single depth scan or image. The key idea is to utilize the fact that predictions from different views of the same or similar objects should be consistent with each other [25]. Wu et al. proposed an end-to-end framework named SK-Net to jointly optimize the inference of Skeypoints via feature learning of a point cloud. These Skeypoints are generated by two complementary regulating losses. A PDE module was designed to extract and integrate the detail feature and pattern feature, allowing local region feature extraction and spatial modeling of a point cloud to be achieved efficiently [26]. Supasorn et al. proposed Keypoint-Net, an end-to-end geometric reasoning framework for learning an ordered set of 3D keypoints; this discovery was guided by carefully constructed consistency and relative pose objective functions [27]. Feng et al. proposed an end-to-end deep network architecture to jointly learn the descriptors for 2D and 3D keypoints from an image and point cloud, establishing 2D-3D correspondence [12]. He et al. studied a novel data-driven method for robust 6DoF object pose estimation from a single RGBD image. Their method is a deep Hough voting network to detect 3D keypoints of objects and then estimate the 6D pose parameters in a least-squares fitting manner [28]. Liu et al. established an easy method for capturing and labeling 3D keypoints on desktop objects with an RGB camera, and developed a deep neural network called KeyPose that learns to accurately predict object poses using 3D keypoints from stereo input; this approach even works for transparent objects [29]. Boyuan et al. presented a framework for learning useful 3D keypoints without supervision for continuous control. The key insight is the leveraging of multi-view consistency with a world coordinate transform in the bottleneck layer in order to learn reliable keypoints [30]. Jakab et al. developed a method for controlling the shape of 3D objects through automatically discovered semantic 3D keypoints and a deformation model learned jointly with the keypoints. The resulting KeypointDeformer model provides users with a simple interface for interactive shape control [31]. Ge et al. proposed a novel approach that directly takes the 3D point cloud of hand as network input and outputs heat maps and unit vector fields on the point cloud that reflect the per-point closeness and directions of the hand joints [32].

Most deep learning-based studies find 3D keypoints from 3D information (depth, point cloud, mesh). On the other hand, our study is different in that it uses a signal processing-based method that estimates the depth of 2D keypoints using the correspondence of 2D keypoints from 2D images. It then generates 3D keypoints by applying the estimated depth to the 2D keypoints.

This paper proposes a new 3D feature extraction method using keypoint-based stereo matching based on a 2D RGB camera with a single lens. Because it estimates depth using keypoints in a 2D RGB image, it does not require additional 3D data to extract 3D keypoints. It proposes a new keypoint-based stereo matching method to overcome the low accuracy of pixel intensity-based stereo matching. Because it requires only 3D keypoints, not depth images, it does not need to calculate disparity with low accuracy for all pixels; that is, the proposed method obtains disparity with high accuracy using the positions of keypoints. By estimating the baseline using a gyro sensor of a mobile device such as a smartphone, it can estimate the actual physical depth. Furthermore, by analyzing the similarity of keypoints between frames, the amount of keypoints transferred and stored can be minimized, and scene changes can be detected. In summary, the technical novelty of our paper can be expressed as follows:

- A new method for 3D keypoint estimation with 3D coordinates from 2D videos without using 3D information such as disparity, depth, 3D mesh, and 3D point cloud;
- A new stereo matching algorithm using the correspondence of a descriptor generated from a SIFT-based 2D keypoint between continuous 2D frames;
- An AR service with security that does not transmit the user’s private and personal image to the server, instead dealing with 2D keypoints that do not contain real feature information;
- Efficient database management and minimized data transmission using 2D keypoint overlapping and scene change detection between continuous frames.

The rest of this paper is organized as follows. Section 2 introduces the basic theory of stereo matching and feature extraction along with relevant prior studies. Section 3 explains the proposed algorithm, describing the entire process and each of its four steps in detail. Section 4 shows the experimental results, and Section 5 concludes the paper.

## 2. Related Works

This paper uses stereo matching and 2D-based feature estimation to extract 3D keypoints using a camera installed in a mobile device. This section explains a new feature estimation algorithm using 2D keypoint-based stereo matching and SIFT.

### 2.1. Stereo Matching

**Figure 1:**
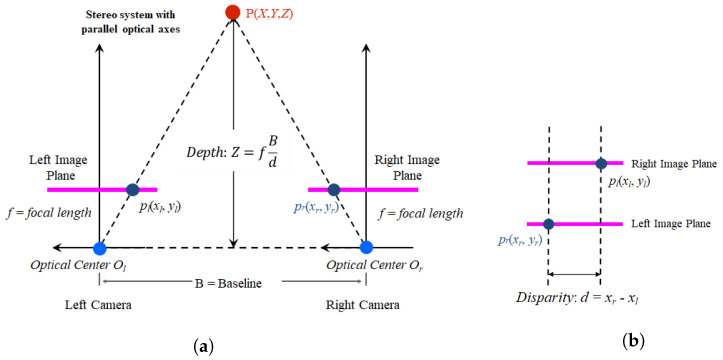
Stereo matching: (**a**) stereo camera configuration and depth definition and (**b**) disparity calculation.

Generally, stereo matching calculates disparity using the relationship between two images (the left and right images), where two photos of the same scene are simultaneously captured by two cameras installed in two different locations. Figure 1a shows a camera setup for capturing a stereo image, and Figure 1b shows the disparity. A pixel (or a region) in the left (reference) image is searched in the right (target) image, with the goal to find a position of an object in the left image in the right image. The horizontal distance ($x_{l}-x_{r}$) between the corresponding pixel (or region) of the two images is defined as the disparity. The disparity is converted to depth using physical information from the capturing equipment, and the 3D position is then estimated from the depth [33,34].

Two cameras in a system for capturing a stereo image are sufficiently calibrated and rectified. However, the camera used in this paper is not aligned physically, and has a different focal length according to the frame. Therefore, in this paper we use keypoint-based rectification, which aligns two corresponding 2D keypoints on an epipolar line. Then, if the disparity is calculated using pixels via stereo matching, the depth can be calculated for the pixels. Although this process seems to be easy work, there are several issues. The most significant problem is the difficulty of finding corresponding pixels with robustness in the two images. Furthermore, although the two cameras are located in a similar vertical position, there are differences in the lighting, lens aperture and exposure, incident illumination, and scene visible to the camera. When an object has a surface with a repeating pattern or high reflective ratio, stereo matching does not provide good results. Several studies have attempted to solve this problem [35]. Recently, the convolutional neural network approach has been introduced to extract the disparity [36,37,38,39]; CNN-based stereo matching tries to extract the disparity in the ill-posed region [40].

### 2.2. Feature Extraction

Feature extraction is a kind of computer vision technology similar to object recognition, image matching, and image synthesis. In feature extraction, finding a robust position is significant without influence about image feature and size, camera viewpoint, or light variant. The well-known earliest method for finding a feature point is the Harris corner detector, which finds a corner point in an image [41]. A corner point is a point that changes rapidly in two or more directions. This method is somewhat weak because it is not robust to changes in the image scale. A complementary method is Mikolajczyk’s Harris Laplacian method [42]. This method finds Harris corner points in various scales and detects robust points for scale variants. Shi and Tomasi proposed the Shi–Tomasi corner considering an affine transformation [43,44]. The most well-known method is Lowe’s SIFT (Scale Invariant Feature Transform) [45]. The SIFT calculates DoG (Difference of Gaussian) in both the scaled and original images and finds points with considerable variance in all resolutions.

**Figure 2:**
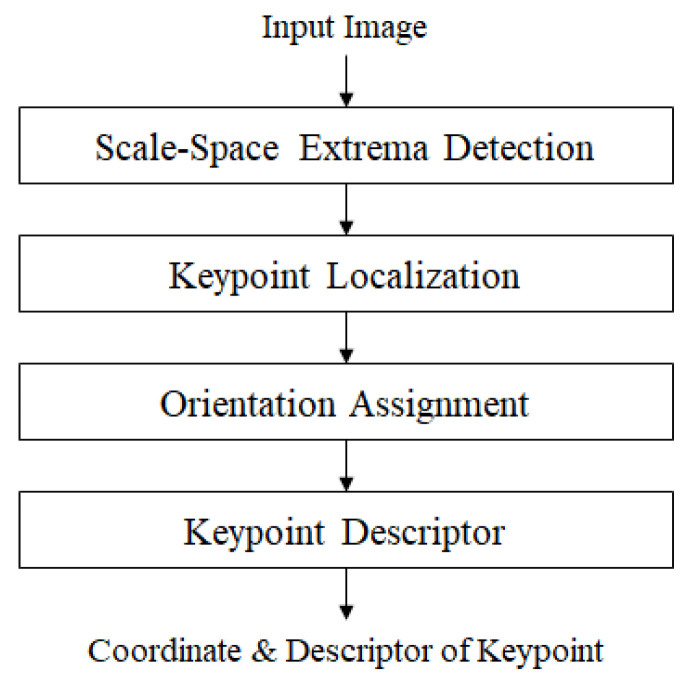
The steps of the SIFT framework.

The SIFT has four steps: scale-space extrema detection, keypoint localization, orientation assignment, and keypoint description, as shown in Figure 2. The scale-space extrema detection step produces keypoint candidates. The image is convolved with Gaussian filters at different scales, then the differences of successive Gaussian-blurred images are taken. Keypoints are then taken as the maxima/minima of the Difference of Gaussians (DoG) that occur at multiple scales. A DoG image D(x,y,σ) is provided in Equation (1):

(1) $$D\left(x,y,\sigma \right)=L\left(x,y,k_{i}\sigma \right)-L\left(x,y,k_{j}\sigma \right)$$

In Equation (1), L(x,y,kσ) is the convolution of the input image I(x,y) with the Gaussian blur G(x,y,kσ) at scale kσ, where σ is the Gaussian blur scale factor.

Scale-space extrema detection produces too many keypoint candidates, some of which are unstable. The next step in keypoint localization is to perform a detailed fit to the nearby data to find the accurate location, scale, and ratio of the principal curvatures. This step consists of three processes: interpolation of nearby data for precise positions, discarding low-contrast keypoints, and eliminating edge responses. The keypoint localization step eliminates those keypoints with poorly determined locations and retains those with high edge responses. In the orientation assignment step, each keypoint is assigned one or more orientations based on local image gradient directions. This step provides invariance to rotation, as the keypoint descriptor can be represented relative to this orientation, thereby achieving invariance to image rotation. Finally, the keypoint descriptor computes a descriptor vector for each keypoint in order to ensure that the descriptor is highly distinctive and partially invariant to the remaining variations, such as illumination, 3D viewpoint, etc. The final step is performed on the image closest in scale to the keypoint’s scale [46,47,48].

This paper extracts keypoints using the SIFT. Keypoints are extracted in each frame captured by a camera installed on a mobile device. If using the SURF [16], the keypoint may be quickly extracted; however, because the accuracy of the keypoints is more important in this paper, we use the SIFT, which has more accuracy than the SURF. The keypoints between two frames (left and right) are searched in each frame using the coordinate of a keypoint and its descriptor. This process enables us to estimate the relationship between the images. The disparity between the two images is calculated from the relationship between the keypoints.

## 3. 3D Feature Extraction

An AR service provides various media services based on the user’s environment after analyzing the environment. Therefore, it is essential for the AR service to analyze the 3D space where the user is located or the 3D object at which the user is looking. In this context, 3D keypoints can be used to provide various user services after space analysis. If a 3D keypoint is detected by the AR service, service within a three-dimensional space is possible. Therefore, studies to create a 3D keypoint for AR service have been conducted, and many seeking to create a 3D point cloud [14]. The most straightforward method for doing this is to estimate the 3D features of images in a server after capturing images using a user’s mobile device and then transmitting them. The 3D keypoint generation technique requires a large amount of calculation. Hence, a server is generally used for processing; the tendency of this approach to use a lot of computing resources to extract 3D features complicate its use in routine services. A mobile device lacks the computing power to estimate 3D features. When transmitting images captured by users to a server, there are network bandwidth problems with increasing data transmission and legal problem around information privacy and security. Therefore, we propose a method for finding 2D keypoints on the user’s device, with other processes implemented by the cloud server.

This section explains the 3D feature extraction algorithm through the method used to estimate 3D keypoints for a 3D object. The 3D feature extraction process consists of four steps: 3D keypoint-based stereo matching, scene change detection, 2D keypoint updating, and 3D keypoint generation. After introducing the structure of the entire algorithm, we explain each step in detail.

### 3.1. Full Process

**Figure 3:**
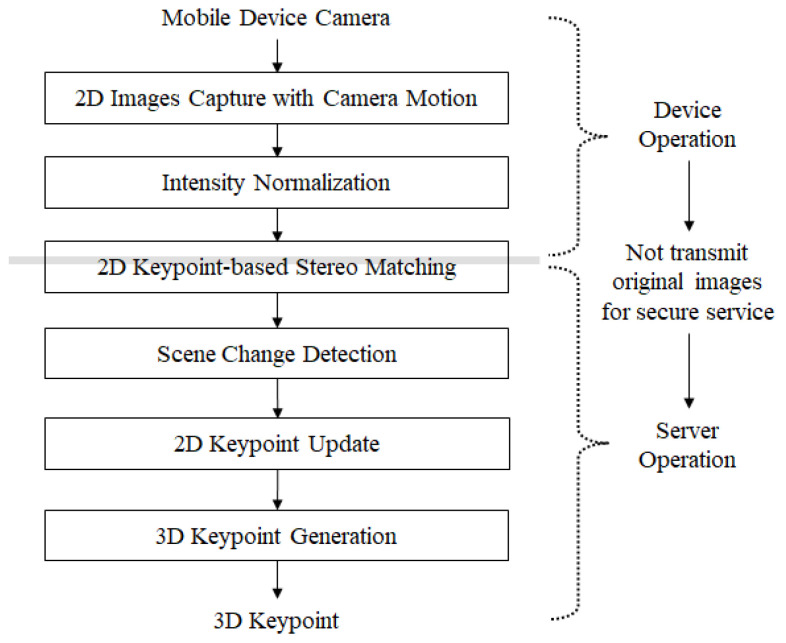
3D Feature extraction algorithm.

The 3D feature extraction step consists of device and server operations. Device operation consists of two steps: 3D image capture and intensity normalization. Figure 3 shows the proposed algorithm for the 3D feature extraction. First, 2D image capture with camera motion captures 2D images according to the movement of a mobile device. This step normalizes the image intensity. Intensity normalization is performed through a simple histogram equalization which enhances the image quality on the side of brightness. Through intensity normalization, dark or bright images are changed to images with a typical intensity scale. Next, 2D keypoints are calculated by stereo matching. This step is carried out on both the device and server. A 2D keypoint generated on the user’s device is transmitted to the server. Finally, the scene change is detected using the relationship between the 2D keypoints of the frames, and the 2D keypoints to be stored are selected by the server.

**Figure 4:**
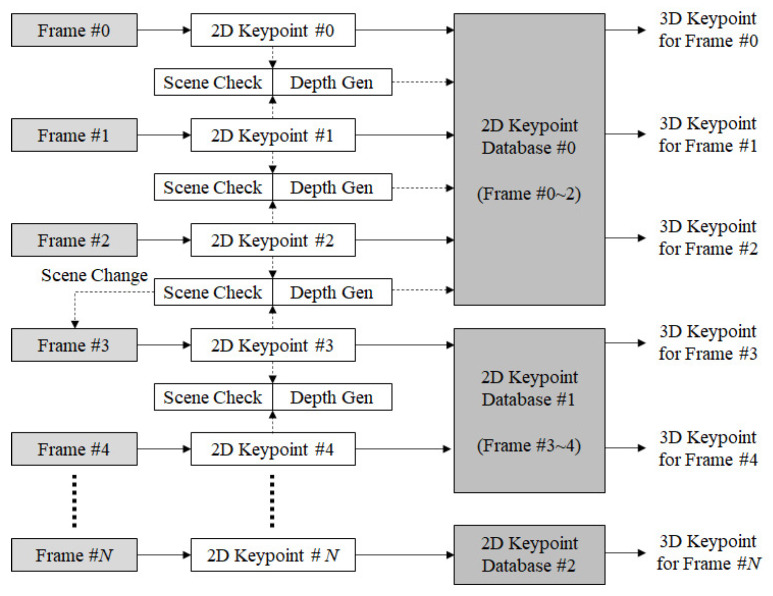
Generation process of 3D keypoints.

Figure 4 depicts the flow of 3D keypoint generation from temporally continuous frames with the order relative to the steps. The 2D keypoints are generated from input images. The scene change is detected by observing 2D keypoints #0 and #1 in frames #0 and #1, respectively, and the depth is calculated using the disparity between the keypoints matched in two adjacent frames, with the moving distance calculated using the output value of the acceleration sensor of a mobile device. When the adjacent frames are regarded as the left and right images, the moving distance of the device corresponds to the baseline distance. When observing frames #2 and #3, if the similarity between the keypoints of the two frames is less than the predefined threshold, a scene change can be considered to have occurred. Thus, the database used to store 2D keypoints is divided, and the keypoints are newly updated.

### 3.2. Keypoint-Based Stereo Matching

**Figure 5:**
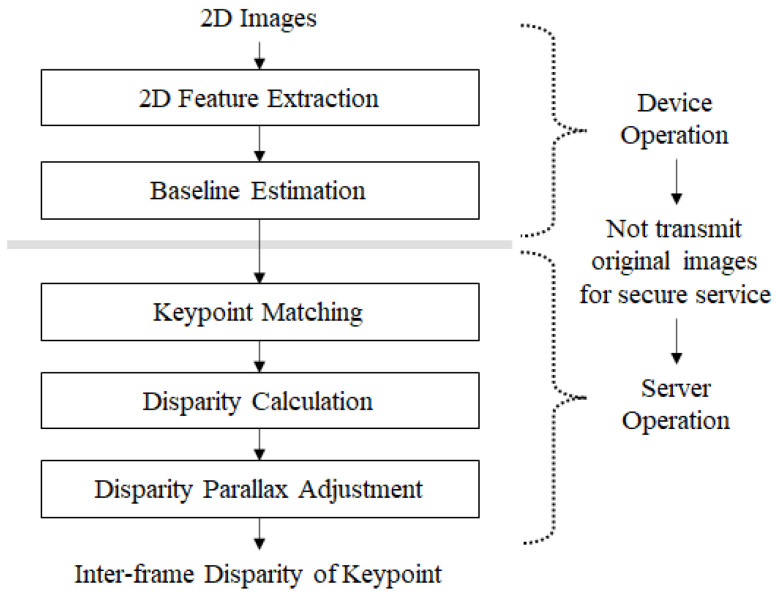
Process of 3D keypoint generation.

The algorithm that generates 2D keypoints based on stereo matching has five steps, as shown in Figure 5. Two continuous frames (left and right) are regarded as stereo images. We use a FLANN-based matching algorithm for stereo matching. Stereo matching as used here has the same meaning as keypoint matching. FLANN stands for Fast Library for Approximate Nearest Neighbors; it contains a collection of algorithms optimized for fast nearest neighbor searching in large datasets and for high dimensional features. It works faster than brute force matching for large datasets. The FLANN-based matcher accepts two sets of options which specify the algorithm to be used and its related parameters [49]. If a device does not move when capturing images, it is hard to find the disparity. If this occurs in keypoint generation, the stored depth is used for the current frame and the operation moves on to the next frame. We explains the algorithm with the assumptions that two frames are input and that they have movement.

**Figure 6:**
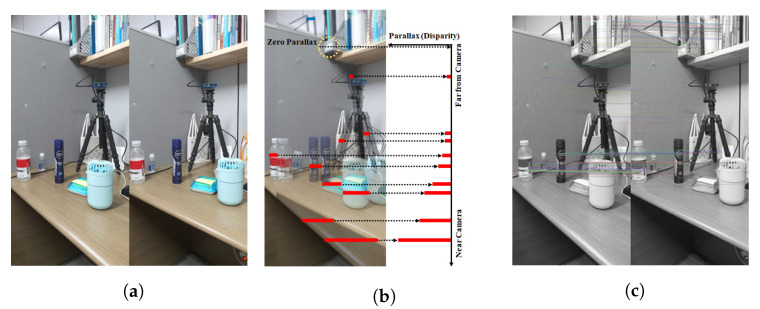
Parallax (or disparity) analysis between stereo images: (**a**) stereo images, (**b**) parallax analysis result, (**c**) keypoint generation.

An example of the operation using two continuous frames is shown in Figure 6 to illustrate the keypoint-based stereo matching process. Figure 6a shows the two continuous frames, Figure 6b shows the disparity between the two frames after overlapping, and Figure 6c shows the result of the corresponding points by 2D keypoints between the two frames.

The disparity is the distance of the *x*-axis between corresponding keypoints in two frames. The disparity is adjusted by the user-defined zero parallax. The adjustment is operated during the calculating process of depth. The equation to calculate disparity for the $n^{th}$ keypoint is defined by Equation (2):

(2) $$D=x_{n+1}-x_{n}$$

### 3.3. Scene Change Detection

There is a similarity between images captured by a moving mobile device. However, if a device moves too fast, the captured images differ. When the difference between the captured images is too significant, this situation is regarded as the occurrence of a scene change. Scene change detection is the process of dividing the database of keypoints based on whether difference between the current image and previous images. If a new scene is captured, the database for the previously stored keypoint cannot be updated with the current information. In this case, a database is newly generated, and the new keypoints are updated in the new database. Next, the 2D keypoints are estimated in the continuous frames, and keypoint matching is executed using the corresponding 2D keypoints. The method for differentiating two frames is by comparing the difference between their 2D keypoints. Finally, the keypoints of the current frame are compared with the previous ones, and the scene change is detected by comparing the result. This relationship is defined as the matching rate in Equation (3). In Equation (3), the number of matched keypoints in the numerator means the number of corresponded (or matched) keypoints between the current frame and the previous frame. The method of matching keypoints between two frames is to compare the descriptor of each keypoint. The number of the current frame’s keypoints in the denominator is the number of all estimated keypoints in the current frame; that is, Equation (3) indicates how many keypoints among the keypoints selected in the current frame existed in the previous frame. We experimentally assumed that scene change occurs in cases of difference over 25%.

(3) $$Matching\;Rate\left(\%\right)=\frac{Number\;of\;Matched\;Keypoints}{Number\;of\;Current\;Frame^{\prime }s\;Keypoints}$$

### 3.4. Keypoint Updating

**Figure 7:**
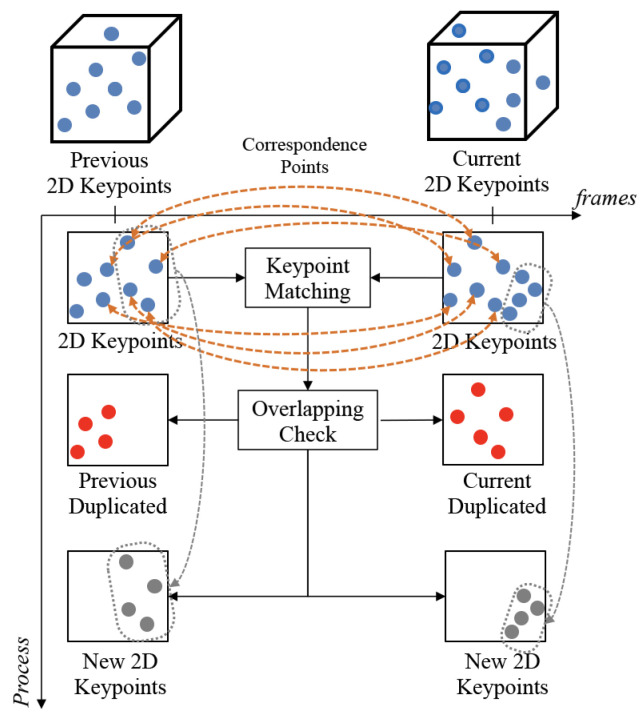
Keypoint matching and overlapped keypoint detect between continuous frames.

As described above, duplicate keypoints may exist between consecutive frames. It is not reasonable to repeatedly process duplicate keypoints in terms of computation and transmission. Therefore, the amount of transmission and storage is significantly reduced by transmitting only newly extracted keypoints, excluding duplicate keypoints. As shown in Figure 7, there are many similarities between continuous frames, and many keypoints overlap. Therefore, all keypoints of the current frame can be estimated if there are only duplicate keypoints of the previous frame and new keypoints of the current frame without storing and transmitting all the keypoints of the existing frame. As shown in Figure 7, duplicate keypoints and new keypoints can be detected through keypoint matching between two frames.

**Figure 8:**
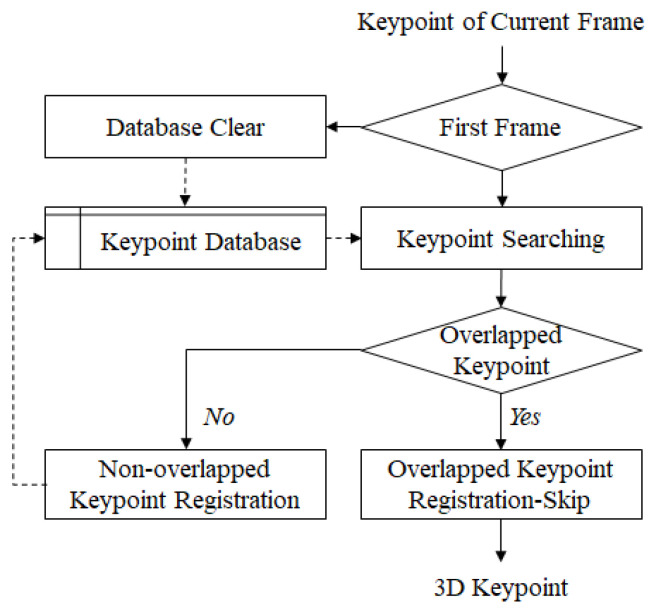
Keypoint update algorithm.

Keypoint updating is used to transmit and store new keypoints of the current frame while not transmitting duplicate keypoints in the present and previous frames. The information of the duplicate keypoints can improve the storage efficiency of the database. In addition, the reduced number of keypoints decreases the amount of calculation required by the unnecessary generation of depth and 3D keypoints. First, the keypoint coordinate and descriptor of the current frame are compared with those of the previous frame. If the same (overlapped) keypoint is detected, the previous keyoint is used and the new one is not stored. Then, the keypoints which exist only in the current frame are processed to generate a depth and 3D keypoint and stored in the keypoint database. Consequently, the keypoint database is updated by only non-overlapped (unduplicated) keypoints. Figure 8 shows the flow chart of the keypoint update algorithm.

**Figure 9:**
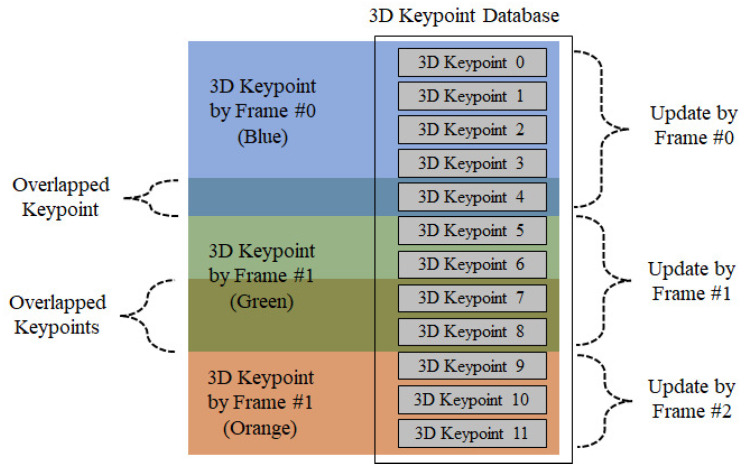
3D keypoint database update.

Figure 9 illustrates the database of the 3D keypoints that are finally saved. As shown in Figure 9, among the 3D keypoints updated by Frame #0, overlapped keypoints are not stored in the database, as they duplicate the keypoints on Frame #1. Furthermore, this relationship is maintained for the subsequent frames. In this way, the information of keypoints and keypoints stored in the 3D keypoint database is minimized.

### 3.5. 3D Keypoint Generation

In this section, we propose a method of converting 2D keypoints obtained from each 2D image frame into 3D keypoints using the relationship between keypoints estimated in each frame. The process of extending a 2D keypoint to a 3D keypoint is as follows. As described above, keypoints make all disparities have negative disparities. Adjusting to the negative disparity is calculated by adding the minimum disparity $d_{min}$ to the original disparity value; $d_{min}$ can be calculated from the relationship between two frames, or it can be calculated in advance using the horizontal resolution of the 2D image. Theoretically, $d_{min}$ can have the maximum width of the horizontal resolution of the 2D image. The *z*-axis of a 2D keypint obtained from the 2D image corresponds to the depth *z* of the 2D keypoint. The depth *z* is generated from the estimated baseline *B* using the parallax value changed in the depth generation, the focal length *f* of the camera, and the acceleration of the mobile device. The process is defined by Equation (2). Next, a 3D keypoint is calculated using the estimated depth and the x and y coordinates of the current frame. Equation (4) is used for the depth value of the 3D keypoint, and the x and y coordinates are normalized to 0∼1.

(4) $$Z=f\times \frac{B}{D-d_{min}}$$

## 4. Experimental Results

The proposed algorithm was implemented using C/C++, OpenCV, OpenMP, and CUDA in Intel I7-7700K CPU @3.6 GHz, 64GB RAM, and 64-bit Windows 10 environment. The image used for the experiment was captured with a Galaxy S10, and the TUM Dataset was used for the data to compare with the actual depth value [50].

Here, various parameters used in this paper are explained. When extracting 2D keypoints, the number of feature points SIFT can output was set to at least 200 million. The octave layer was set to 3, the contrast threshold to 0.04, the edge threshold to 10, and the sigma to 1.6. Next, diagonal matching was considered an error in the process of matching the descriptor, and they were removed. In the keypoint-based stereo matching, if the matched two keypoints had a difference of 200 pixels or more in the *x* and *y* axes, this was considered error matching. All of these parameters were obtained experimentally.

### 4.1. Baseline Calculation

**Figure 10:**
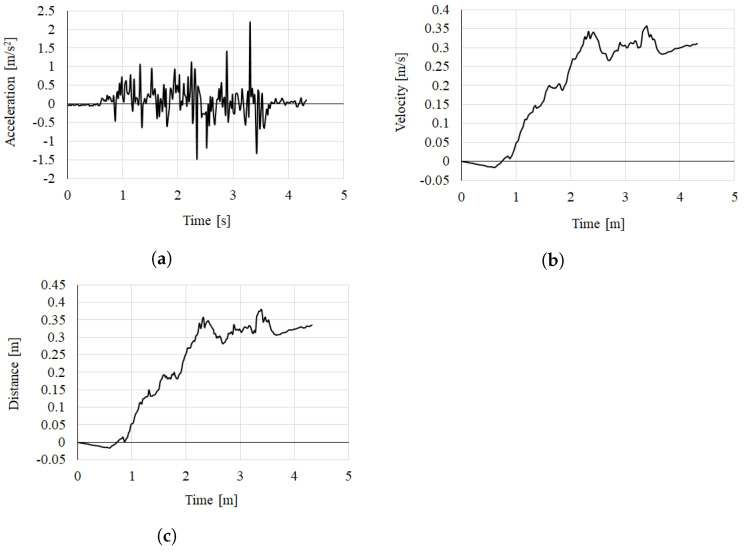
Distance estimation result of the baseline: (**a**) accelerating value from the gyrosensor, (**b**) speed, and (**c**) estimated distance.

The baseline represents the actual distance traveled by the camera. In general, stereo cameras have a fixed baseline. In this paper, the baseline is estimated by measuring the acceleration value of the gyrosensor according to the movement of the camera. Acceleration values were obtained while moving the camera for 4.32 s, and the actual moving distance of the mobile device was measured by 45.73 cm using a laser range finder. Figure 10a shows the acceleration obtained from the gyrosensor, Figure 10b the velocity, and Figure 10c the estimated distance. The distance obtained through acceleration showed a total movement of 45.11 cm, and the error that occurred was 0.62 mm. These results verified that the distance of the mobile camera estimated using the acceleration measured from the gyrosensor can be used to estimate the baseline.

### 4.2. Result of Keypoint-Based Stereo Matching

**Figure 11:**
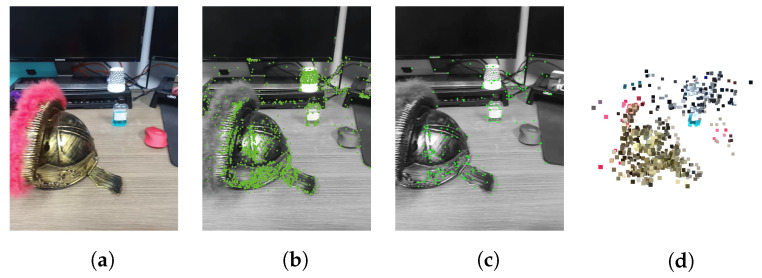
Keypoint-based stereo matching result: (**a**) RGB image, (**b**) keypoints of the previous frame (the left image), (**c**) keypoints of the current frame (right image), (**d**) 3D keypoints result plotted in a 3D space with RGB information.

In this paper, we use the rear camera of Galaxy S10 with a focal length of 53.6 mm. The baseline distance was calculated using the acceleration obtained through the accelerometer of the same mobile device. We use an Intel RealSense Depth Camera D415 to verify the estimated depth. To match the structure of this camera, the baseline was set to 55 mm. Then, keypoint-based stereo matching was performed using the keypoint of the first frame and the frame after moving 55 mm. Figure 11a shows the captured RGB image, and Figure 11b shows the keypoint estimation result in the previous frame. Figure 11b shows the keypoint estimation result in the current frame taken after moving the camera by 55 mm. Figure 11d shows the 3D keypoint generated by keypoint-based stereo matching.

### 4.3. Keypoint Update Result

**Figure 12:**
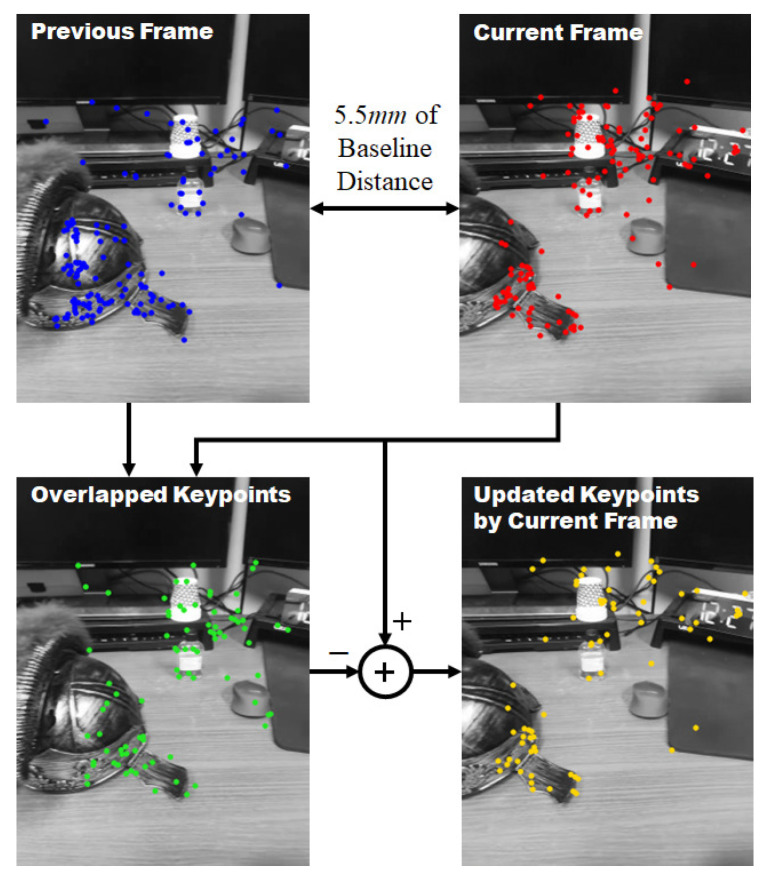
Keypoint update algorithm: keypoint information of the first frame (left top), keypoint information after 55 mm movement (right top), matched keypoint information (left bottom), and newly generated keypoint information (right bottom).

After extracting the keypoint of the input image according to the movement of the camera and matching it with the keypoint of the previous frame, a new keypoint is added to the keypoint database by excluding the duplicated keypoint in the two images. Figure 12 shows the results of this process. The keypoint result extracted from the first frame and the keypoint result after the mobile device moved as much as the 55 mm baseline are shown in the upper part of Figure 12. When the descriptors of the keypoints of the two frames are matched, updated keypoints for the current frame are obtained if duplicate keypoints are excluded from matching keypoints.

Using five frames, we verified how the keypoint update algorithm quantitatively contributes to the reduction of keypoint storage. The first frame stores all keypoints as the start frame. From the next frame onwards, keypoints that overlap with the previous frame are not saved, and only new keypoints are saved. When the second frame is input, only 220 keypoints are updated, and 645 duplicate keypoints are not stored in the database. After comparing the third frame with the second frame, 754 keypoints are not saved and 221 new keypoints are updated. When 2799 keypoints were matched for five frames (excluding the start frame), the average number of keypoints to be updated was only 27.67%. Table 1 summarizes the ratio of overlapped and updated keypoints for each frame in five frames.

### 4.4. Result of Scene Change

**Figure 13:**
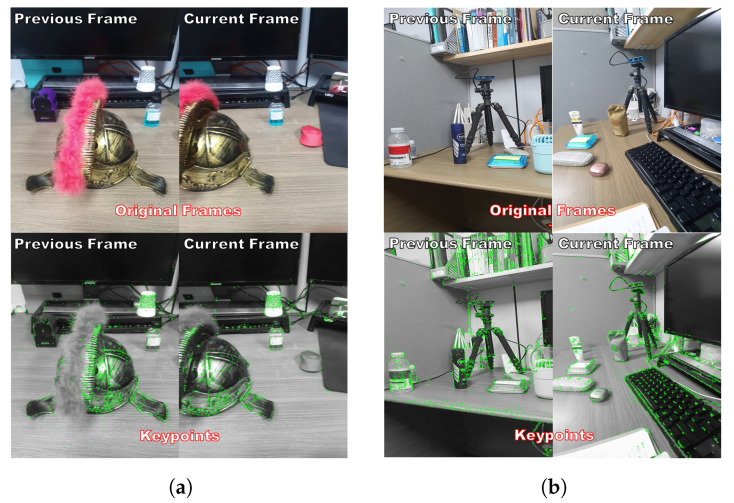
Scene change detection result: (**a**) 74.57% overlapped keypoints, (**b**) 5.76% overlapped keypoints, indicating a scene change.

An example of the results of scene change detection is shown in Figure 13. Figure 13a shows a case where scene change does not occur, and Figure 13b shows the result when a scene change occurs. In the two figures, the upper two figures are the original RGB images, and the lower two figures are the keypoint extraction results. In Figure 13a, the overlapped keypoint between the previous and current frames is 74.57%, a result that can be considered as no scene change. In Figure 13b, the overlapped keypoint between the previous and current frames is 5.76%, a result that can be considered a scene change. When visually confirming Figure 13a, it seems that the change between the two frames is large; however, there was no significant change in the keypoints. Figure 13b shows the same scene shot from different angles. However, if the characteristics of the keypoint descriptor are different depending on the angle, it may be judged to be a different scene. Because this paper proposes a method for obtaining keypoints, the similarity of the descriptors of the keypoints is more important than the visual similarity.

### 4.5. Comparison of Results with TUM Dataset

**Figure 14:**
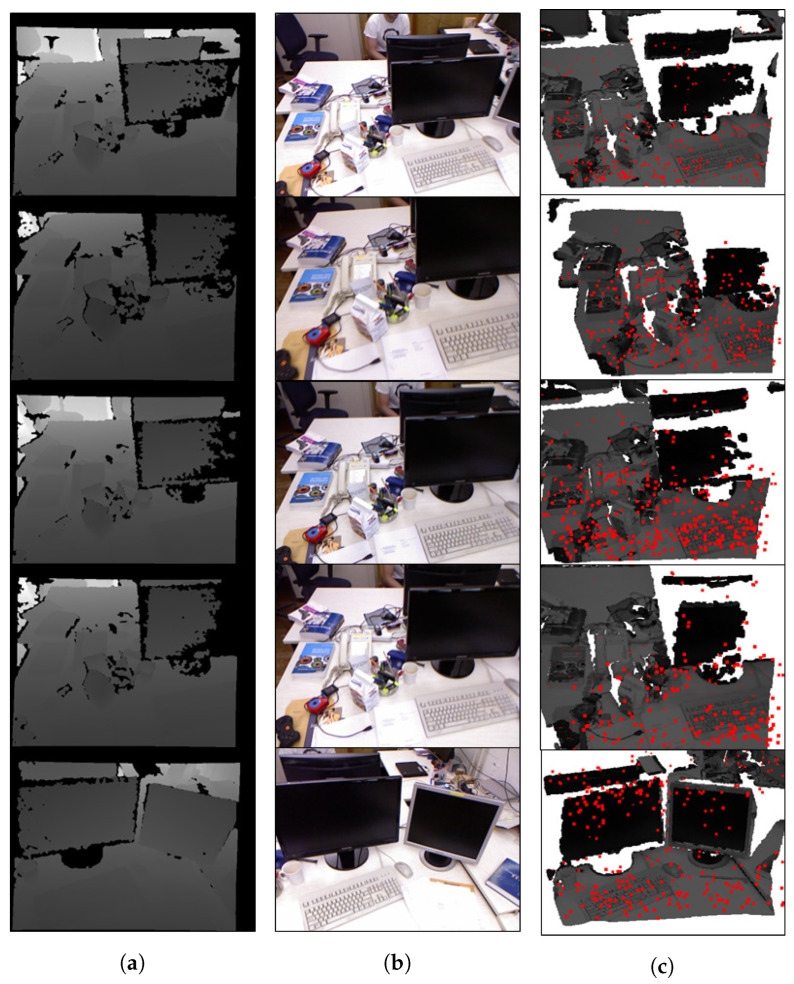
TUM dataset: (**a**) depth map, (**b**) RGB, (**c**) point cloud and 3D keypoints.

This experiment compares the proposed method with data from the TUM Dataset and checks whether the three-dimensional keypoints extracted by the proposed algorithm are similar to the true depth value. In the experiment, 3D keypoints were extracted with our algorithm using the focal length and gyro sensor values of the TUM dataset. The depth images of the TUM dataset are shown in Figure 14a, and the RGB images are shown in Figure 14b. Figure 14c plots the results of the 3D keypoint against the result when converting the TUM Dataset to the original point cloud.

Table 2 shows the coordinates between the 3D keypoint generated by the proposed algorithm and the ground truth of the original point cloud from the TUM dataset as a result of obtaining the Euclidean distance of the original coordinates. In actual 3D space, the maximum Euclidean distance between frames was 16.32 mm and the minimum distance was measured to be less than 0.03 mm. The average maximum Euclidean distance in five frames had a difference of 13.00 mm, and the overall keypoint had an average difference of 5.98 mm compared to the original. This confirms that the appropriate depth can be estimated by stereo matching using the baseline obtained by estimating the coordinates of the 3D keypoint.

### 4.6. Performance Comparison with Previous Study

This paper estimates 3D keypoints from 2D images. The actual distance difference between the 3D keypoint information generated from the 2D image and the 3D point cloud of the ground truth was calculated, and the estimated keypoint was validated. Because the point cloud *x*, *y*, and *z* coordinates of the ground truth for the 3D keypoint are matched to the 2D feature point, there is no difference in coordinates between 2D and 3D. To maintain 2D–3D correspondence, 2D–3D MatchNet [12] was used to project ISS (Intrinsic Shape Signatures) keypoints to all images in the view. Next, the nearest neighbor of the SIFT keypoint in each image was found; the keypoint was considered valid if it was within three pixels. The 3D keypoint extracted from the proposed algorithm has an average difference of 5.98 mm from the original ground truth. In the TUM dataset, the difference of three adjacent pixels in the 2D image corresponds to an average error of 15 mm in the 3D point cloud of the ground truth. In our results, all keypoints were within three pixels in 2D space. Most previous studies estimating 3D keypoints use 3D meshes, depth images, and 3D point clouds as inputs. These use 3D information to find 3D keypoints in 3D space. Because most studies have different input domains and methods of defining 3D keypoints, the comparison of results between them may be somewhat limited. In addition, because our study is a method for finding 3D keypoints in 2D videos, there may be limitations when comparing the results with such studies.

The results of our study and previous studies were compared using AE (Average Error) and PAE (Pose-invariant Distance Metric) [25]. Because certain experimental conditions and environments were different, it is difficult to determine the superiority of each method solely by comparing these results. The AE can provide the relationship between each predicted keypoint configuration and the corresponding annotation, and the PAE can offer a new metric [25]. The AE and PAE are shown in percentages, and represent the relative ratio to the diagonal length of the 3D bounding box. Our method is rule-based, while all other methods are deep learning-based. Table 3 compares the results. In the results, although the difference in error is very small, the result with our proposed method showed the lowest error. In Table 3, the default method uses the vanilla ResNet [51] based on the method proposed by [25], while the ADDA method uses the generative adversarial network based on [25].

### 4.7. Ablation Study

We performed an ablation study to evaluate each component of our approach. We dealt with three ablation studies. The first is for the processing time in Section 4.7.1, the second is for the searching range in Section 4.7.1, and the third is for the baseline distance. Section 4.7.1 shows how the processing time changes depending on whether keypoint updating and scene change detection are included. Section 4.7.2 shows the degree to which the accuracy of the corresponding point varies depending on whether the search range is set using 2D keypoint-based stereo matching. We obtained the highest accuracy experimentally using a setting around 200 pixels. In Section 4.7.3, we present the experimental results on the accuracy of 3D keypoints depending on whether the baseline distance was set.

#### 4.7.1. Processing Time

**Figure 15:**
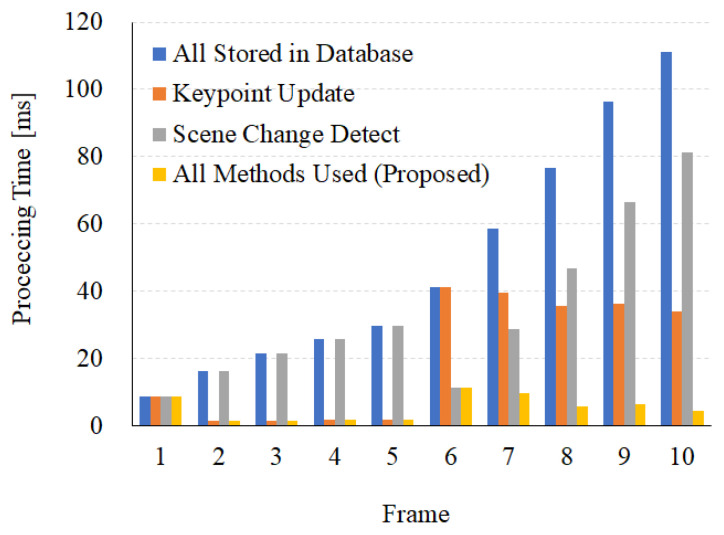
Processing time reduction through scene change detection and duplicate keypoint removal.

The processing time required to calculate a new 3D keypoint was observed for the keypoint update and scene change algorithm. The experiment was conducted using a sequence with ten frames, with a scene change in the 6th frame. Ifthe keypoints are updated without the keypoint update and scene change algorithm, all new keypoints are stacked in the database. Furthermore, when a new scene starts, new keypoints are matched with the previous ones with totally different features. The first frame has a processing time of 8.68ms. The calculation time for five frames is 15.03 ms and 101.86 ms during the scene change when using and not using the keypoint update algorithm, respectively. After the scene change, the processing time is 37.4 ms in both cases. If all keypoints are stored in the database, it takes 383.68 ms. When using the keypoint update algorithm, it takes 186.75 ms. When the database is initialized and divided by the scene change algorithm, it takes 11.43 ms at the 6th frame and 234.45 ms for the other frames. The processing times for ten frames are 383.68 ms, 186.75 ms, 234.35 ms, and 37.41 ms. These results translate to a performance enhancement of 10.25 times. Figure 15 shows the processing times in the case of not using the keypoint update and scene change, the case of only using the keypoint update, the case of only using the scene change, and the case of using all algorithms.

#### 4.7.2. Search Range

**Figure 16:**
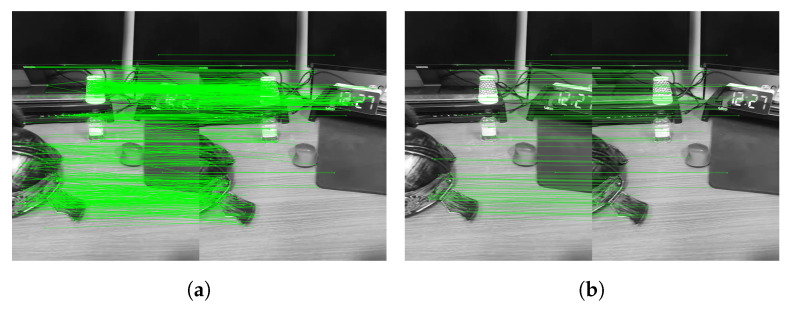
Estimation result of corresponding points (**a**) without search range and (**b**) with search range of 200 pixels.

In 2D keypoint-based stereo matching, we experimented with using a search range. Considering the movement distance per frame of the mobile device, performing stereo matching over a limited distance may cause an error. Because stereo matching may cause errors in the estimation process, the probability of generating errors should be reduced as much as possible. If the search range is extensive, more errors may occur by performing many checks on unnecessary positions. Through experiments, we found that 200-pixels is the most suitable search range. Figure 16a shows the result of performing 2D keypoint-based stereo matching without limiting the search range. As can be seen from the figure, the stereo matching result includes many errors. Figure 16b shows the result when the search range is limited to 200 pixels or less. In this case, relatively few errors occur. In addition, in comparison with Figure 16a the corresponding point in the diagonal direction does not occur.

#### 4.7.3. Baseline Distance

**Figure 17:**
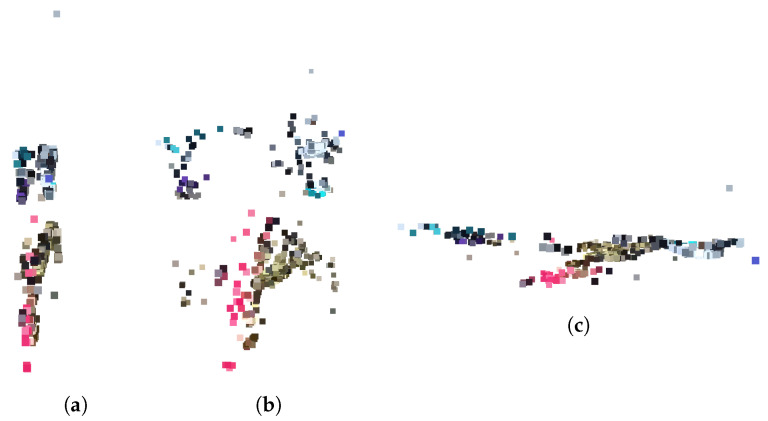
3D keypoint results according to baseline length (top-view) of (**a**) 10 mm, (**b**) 55 mm, and (**c**) 150 mm.

Setting the baseline is very important when converting the estimated disparity into depth. It is necessary to generate the *z*-axis coordinates of the 3D keypoint as well. We conducted an experiment according to the method of setting the baseline. Based on the experimental results, the baseline in our experiment was fixed at 55 mm. The experiment was performed to check how the 3D keypoint is positioned in space. For this experiment, 3D keypoints were extracted while adjusting the length of the baseline in three ways. Figure 17a shows the result when the baseline is 10 mm, Figure 17b shows the result when the suggested distance is 55 mm, and finally, Figure 17c shows the result when the baseline is 150 mm. Observing the three results, correct 3D keypoints were not generated when an appropriate baseline was not set. In the case of Figure 17a,c, the space was compressed and severe distortion occurred. In the case of Figure 17b, the space was normally formed by the 3D keypoint.

## 5. Conclusions

This paper proposes a 3D keypoint extraction method using a single mobile device. 3D keypoints were extracted using a monocular camera and keypoint-based stereo matching. Using the keypoint update algorithm for 3D keypoint generation improves the amount of computation and storage required for the database. In addition, scene change detection was performed using the keypoint matching rate. Finally, we verified whether the proposed keypoint extraction method is valid. Through comparison with actual depth values from the TUM dataset, it was confirmed that the proposed method correctly expresses 3D information. As a follow-up study, we intend to use a keypoint extracted by the proposed algorithm with object recognition algorithms.

## Figures and Tables

**Table 1:** The ratio of overlapped and updated keypoints between frames.

Frame	Overlapped Ratio		Updated Ratio		Total
1	-	0%	1048	100%	1048
2	645	74.56%	220	25.44%	865
3	754	77.33%	221	22.67%	975
4	394	72.70%	148	27.30%	542
5	270	64.74%	147	35.26%	417
Average	515.75	72.33%	184	27.67%	700

**Table 2:** Comparison of Euclidean distances between the 3D keypoints of the proposed algorithm and the TUM dataset (unit: mm).

	1	2	3	4	5	Total
Min	0.11	0.11	0.04	0.03	0.10	0.07
Max	16.32	12.56	10.21	11.33	14.62	13.00
Average	7.45	6.10	5.78	4.37	6.21	5.98

**Table 3:** Comparison of results between previous studies and our method [25].

Target Metric	AE	PAE
Default [51]	27.59	13.44
ADDA [52]	26.16	12.31
Supervised	11.96	7.67
Unsupervised	25.24	11.38
Ours	11.42	7.60

## Data Availability

Not applicable.
